# Consumption-like Thrombocytopenia Phenotype Predicts ICU Mortality with Particular Relevance in Patients with Malignancy: A Retrospective Single-Center Cohort Study of 2188 Patients

**DOI:** 10.3390/jcm15124720

**Published:** 2026-06-17

**Authors:** Tuba Güllü Koca, Fazıl Çağrı Hunutlu, Ayşegül Ertınmaz, Ahmet Mert Yanık, Yusuf Bilen, Nizameddin Koca

**Affiliations:** 1 Department of Hematology, Bursa City Training and Research Hospital, University of Health Sciences, Bursa 16280, Turkey; tubakocamd@gmail.com (T.G.K.); fazilhunutlu@gmail.com (F.Ç.H.); ahmetmertyanik@gmail.com (A.M.Y.); bilenyusuf@hotmail.com (Y.B.); 2 Department of Internal Medicine, Bursa City Training and Research Hospital, University of Health Sciences, Bursa 16280, Turkey; aertinmaz@yahoo.com

**Keywords:** thrombocytopenia, intensive care unit, mean platelet volume, platelet phenotype, mortality, malignancy, critical illness

## Abstract

**Background:** Thrombocytopenia develops in roughly one in three intensive care unit (ICU) patients and is associated with worse outcomes. Most prognostic studies treat it as a single entity defined by platelet count (PLT) alone. We examined whether classifying the dominant pathophysiological direction (peripheral consumption versus impaired production), using admission mean platelet volume (MPV), adds prognostic information beyond the count itself. **Methods:** We analyzed 2188 consecutive adults admitted to a tertiary medical ICU between 2019 and 2023. Patients were classified as: normal platelet count (PLT ≥ 150 × 10^9^/L), consumption-like thrombocytopenia (PLT < 150 × 10^9^/L with elevated MPV), or production-like thrombocytopenia (PLT < 150 × 10^9^/L with normal or low MPV). The primary outcome was all-cause ICU mortality. Multivariable logistic regression adjusted for age, APACHE II score, albumin, creatinine, and malignancy. A prespecified malignancy subgroup analysis included a phenotype interaction term. **Results:** Of 2188 patients (median age 70 years; 57.6% male), 717 (32.8%) had thrombocytopenia; of these, 703 with available MPV were classified: 249 consumption-like and 454 production-like; the three groups were similar in age and sex distribution (*p* = 0.002 for age; *p* = 0.876 for sex). Active malignancy was present in 585 patients (26.7%) and was more frequent in the consumption-like group (34.5% vs. 25.2% PLT-normal; *p* = 0.008). Overall ICU mortality was 44.4% and differed across phenotypic groups (52.0% consumption-like vs. 46.7% production-like vs. 42.4% PLT-normal; *p* = 0.010). In the full cohort, consumption-like thrombocytopenia was independently associated with ICU death (OR 1.46, 95% CI 1.08–1.99; *p* = 0.016); production-like was not (OR 1.12; *p* = 0.302). In patients with malignancy, the adjusted OR was 2.48 (95% CI 1.37–4.48; *p* = 0.003); a formal interaction test was significant [interaction OR 2.12 (95% CI 1.08–4.15; *p* = 0.029)]. Among patients without malignancy, no association was found (OR 1.18; *p* = 0.397). **Conclusions:** Classifying thrombocytopenic ICU patients by mechanism rather than count alone identified a higher-risk subgroup. Consumption-like thrombocytopenia was independently associated with ICU death; production-like was not. The association was substantially stronger in patients with malignancy. The classification requires only the standard blood count and adds no testing burden.

## 1. Introduction

Thrombocytopenia, defined as a platelet count below 150 × 10^9^/L, develops in 20–50% of ICU patients and is associated with clinical deterioration [1,2]. Its association with mortality, prolonged ventilation, and transfusion is well established [1,2,3]. What remains less clear is whether the cause of the low count matters as much as the count itself.

The distinction between consumption and impaired production has direct clinical implications. Anticoagulation or plasma exchange may help a patient with sepsis-driven DIC-consuming platelets; the same treatment in a patient with marrow suppression could cause serious harm. Yet most prognostic studies and most bedside monitoring focus on the platelet nadir or its trajectory, without classifying the underlying mechanism [4,5,6,7,8,9].

MPV appears on every standard blood count and reflects platelet size and thrombopoietic activity. When platelets are destroyed at the periphery, the marrow responds by releasing large, immature platelets, which raise MPV. When the marrow itself is failing, output is smaller, and MPV stays low [10]. This physiological pattern forms the basis of a simple two-group classification of thrombocytopenic patients: consumption-like (high MPV) and production-like (normal or low MPV).

Whether this classification predicts ICU mortality independently of severity scores has not been tested in a large cohort. The question may be especially relevant in cancer patients. Thrombocytopenia predicts ICU admission and death in hematologic malignancy, yet published studies have examined platelet count rather than its mechanistic basis [11]. In cancer, consumption-dominant thrombocytopenia can arise through tumor-driven DIC, procoagulant microparticles, or paraneoplastic platelet activation. Whether elevated MPV identifies this pattern in the oncological ICU setting has not been studied.

We therefore conducted a retrospective cohort study of 2188 medical ICU admissions. Our primary question was whether consumption-like thrombocytopenia predicts all-cause ICU death after adjustment for severity scores, and whether this association is modified by malignancy status. Secondarily, we examined whether a formal interaction test supports effect modification and whether the platelet index profile (PDW, P-LCR, PCT) is consistent with the biological validity of the MPV-based phenotyping.

## 2. Methods

### 2.1. Study Design and Setting

We conducted a single-center retrospective cohort study at Bursa City Training and Research Hospital, a tertiary academic medical center in Turkey. All consecutive adults (age ≥ 18 years) admitted to the medical ICU between January 2019 and May 2023 were eligible. The study was conducted in accordance with the principles of the Declaration of Helsinki. The institutional ethics committee approved the study (Bursa City Training and Research Hospital Clinical Research Ethics Committee, approval number: 2023-10/13, date: 7 June 2023). The requirement for individual informed consent was waived by the Bursa City Training and Research Hospital Clinical Research Ethics Committee on the grounds that the study used only routinely collected, retrospective clinical data; no additional procedures, interventions, or patient contact were involved; and patient data were analyzed in anonymized form with no risk of harm to participants.

### 2.2. Patient Population

Patients were included if complete hemogram data (specifically platelet count and MPV) were available at ICU admission. The only exclusion criterion was primary admission for acute trauma. Of 2188 patients meeting these criteria, all were included.

### 2.3. Platelet Phenotype Classification

We classified thrombocytopenia as consumption-like or production-like based on admission MPV. Patients with PLT ≥ 150 × 10^9^/L formed the reference group. Among thrombocytopenic patients (PLT < 150 × 10^9^/L), those with MPV above the 75th percentile of the non-thrombocytopenic population (11.3 fL) were classified as consumption-like; the remainder were classified as production-like. Complete blood counts were performed on a Sysmex XN 9100 hematology analyzer (Sysmex Corporation, Kobe, Japan); EDTA-anticoagulated samples were analyzed within 4 h of venipuncture, consistent with established pre-analytical recommendations for MPV stability.

The physiological basis for this approach is straightforward. Peripheral platelet destruction triggers compensatory thrombopoiesis, releasing large, metabolically active platelets and raising MPV. Bone-impaired marrow production results in reduced platelet output and a lower or normal MPV [10]. These groups are not intended to represent confirmed diagnoses but rather to capture the dominant pathophysiological direction at the time of admission.

### 2.4. Data Collection

Baseline data collected from medical records included demographics, eight comorbidities (diabetes, hypertension, chronic kidney disease, coronary artery disease, COPD, prior stroke, heart failure, and malignancy), primary admission diagnosis, and admission laboratory values: complete blood count indices, CRP, LDH, albumin, creatinine, APACHE II score, and GCS score. Malignancy type was classified as solid or hematological.

### 2.5. Outcomes

The primary outcome was all-cause ICU mortality. Secondary outcomes were the ability of platelet indices (MPV, PDW, P-LCR, PCT) to distinguish between the two thrombocytopenic phenotypes, and phenotype-specific mortality within six prespecified clinical subgroups: malignancy, acute kidney injury, sepsis, gastrointestinal bleeding, COVID-19, and chronic kidney disease.

### 2.6. Statistical Analysis

Continuous variables were assessed for normality by visual inspection of histograms and Q-Q plots and by the Shapiro–Wilk test; all key variables showed right-skewed distributions. They are therefore reported as median (IQR) and compared with the Kruskal–Wallis test. Pairwise post hoc testing used Mann–Whitney U with Bonferroni correction (adjusted alpha 0.0167). Categorical variables are reported as *n* (%) and compared using the chi-square test; pairwise comparisons are performed using Fisher’s exact test. Effect sizes for continuous variables were reported as rank-biserial correlations (r; |r| > 0.5 considered large). Categorical associations used Cramér’s V.

### 2.7. Sample Size

This retrospective study used all consecutive eligible admissions over the study period (*n* = 2188); no a priori sample size calculation was performed. As a post hoc assessment, detection of an odds ratio of 1.46 for consumption-like thrombocytopenia (observed prevalence 11.4%) with 80% power at a two-sided α = 0.05 requires approximately 860 events. The cohort provided 960 deaths in the full cohort and 871 in the analytic dataset (*n* = 1954), indicating adequate power for the primary endpoint. Multivariable logistic regression modeled ICU death as a function of age, APACHE II, albumin, creatinine, CRP, LDH, malignancy, and platelet phenotype (consumption-like and production-like vs. PLT-normal). GCS was initially considered a covariate but excluded from the final model because APACHE II incorporates the neurological component of GCS (scored as 15-GCS); including both produced a multicollinearity-driven suppressor effect that reversed the direction of GCS and inflated its coefficient without improving model fit. The impact of this exclusion on the primary estimate was negligible (consumption-like OR change < 0.01). Model discrimination was assessed by the area under the receiver operating characteristic curve (AUC). Model fit was assessed by the Akaike Information Criterion (AIC), Bayesian Information Criterion (BIC), and the Hosmer–Lemeshow goodness-of-fit test (10 groups).

A prespecified subgroup analysis in patients with malignancy additionally adjusted for hematological versus solid malignancy type. In a prespecified sensitivity analysis, we tested alternative MPV thresholds for phenotype classification: the 50th percentile (10.5 fL) and the institutional upper reference limit (12.0 fL). Results are presented in Supplementary Table S1. All analyses were performed in R (version 4.4.1; R Core Team, 2024). Missing data for all primary analysis variables are summarized in Supplementary Table S2. Descriptive statistics and group comparisons were used with the tableone package. Multivariable logistic regression used base R glm() with profile-likelihood confidence intervals (broom package). Model discrimination was assessed by the area under the receiver operating characteristic curve (pROC package). ROC analysis of P-LCR used the pROC package with Youden’s J criterion for cut-off selection. Survival analysis used the survival and survminer packages. A two-sided *p* < 0.05 was considered statistically significant.

In a prespecified sensitivity analysis, we examined whether five hemogram-derived inflammatory indices, neutrophil-to-lymphocyte ratio (NLR), lymphocyte-to-monocyte ratio (LMR), platelet-to-lymphocyte ratio (PLR), red cell distribution width (RDW-CV), and systemic immune-inflammation index (SII), were independently associated with ICU mortality, and whether adding NLR and RDW to the primary model altered the consumption-like phenotype estimate. Each index was compared between survivors and non-survivors by the Mann–Whitney U test with rank-biserial correlation as the effect size, and across phenotype groups by the Kruskal–Wallis test with Bonferroni-corrected pairwise comparisons (adjusted alpha 0.0167). For the sensitivity regression, NLR and RDW were added to the primary model; both models were fitted on the same complete-case dataset (*n* = 1939) to permit a valid likelihood ratio test (lmtest package). Results are in Supplementary Table S3 and Figure S1.

### 2.8. APACHE II Calibration Analysis

To contextualize the cohort’s overall mortality, we calculated APACHE II-predicted mortality for each patient using the standard medical non-operative logistic regression formula (logit = −3.517 + 0.146 × APACHE II score) and derived the standardized mortality ratio (SMR = observed mortality/predicted mortality) for the overall cohort, by APACHE II score category (<10, 10–14, 15–19, 20–24, 25–29, ≥30), and separately for patients with and without active malignancy. The 95% confidence interval for the SMR was derived using the Poisson approximation. Full results are presented in Supplementary Table S4.

## 3. Results

### 3.1. Patient Characteristics

**Figure 1:**
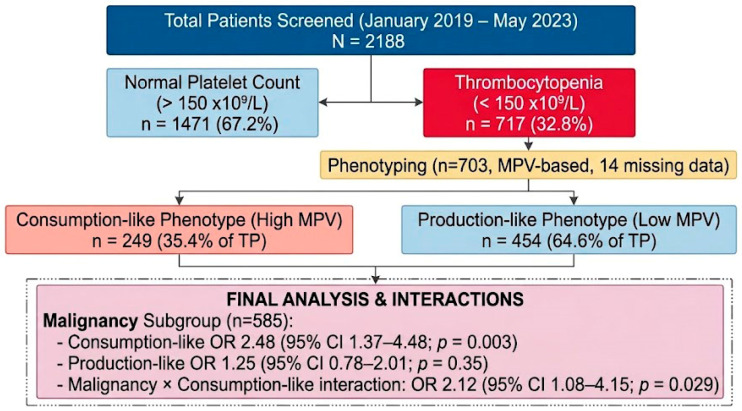
Study flow diagram. MPV = mean platelet volume; OR = odds ratio; CI = confidence interval.

The cohort included 2188 patients (Table 1; Figure 1). Median age was 70 years; 57.6% were male. Overall ICU mortality was 44.4%. Of the cohort, 1471 (67.2%) had normal platelet counts, 249 (11.4%) had consumption-like thrombocytopenia, and 454 (20.7%) had production-like thrombocytopenia. Acute kidney injury was the most common admission diagnosis (26.9%), followed by pneumonia (21.6%) and gastrointestinal bleeding (9.7%). Malignancy was present in 585 patients (26.7%). Median APACHE II score was 18. APACHE II-predicted mortality was 37.5%, yielding an overall SMR of 1.18 (95% CI 1.16–1.21). Excess mortality was concentrated among patients with active malignancy (SMR 1.49), while non-malignant patients had an SMR close to 1.0 (SMR 1.06), consistent with reasonable APACHE II calibration in non-malignant patients. The markedly elevated SMR in the APACHE II < 10 stratum (6.24) reflects a known limitation of APACHE II in this population: patients with low physiological derangement at ICU admission but high background mortality risk, including those with advanced malignancy, dementia, or a palliative trajectory, tend to receive low APACHE II scores despite a high likelihood of in-hospital death. Detailed calibration results by APACHE II category and malignancy status are presented in Supplementary Table S4.

The three groups were similar in sex distribution, comorbidity burden, APACHE II score, and GCS (all *p* > 0.10). The one notable imbalance was malignancy prevalence: higher in the consumption-like group (34.5%) than in the PLT-normal (25.2%) or production-like (27.5%) groups (*p* = 0.008).

### 3.2. Platelet Indices Across Phenotypic Groups

**Figure 2:**
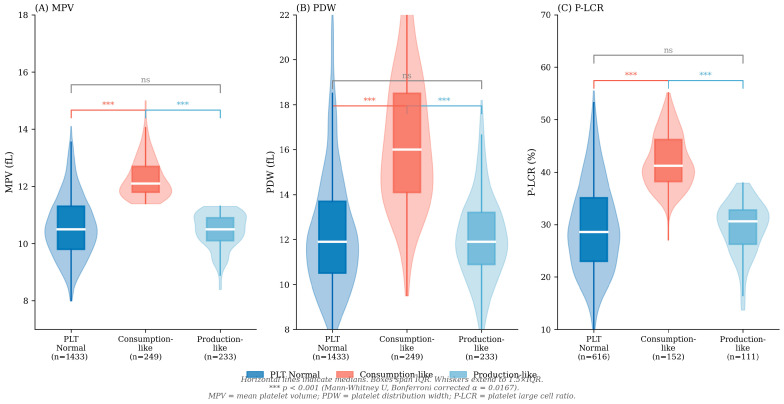
Distribution of platelet indices by phenotypic group. Horizontal lines = medians; boxes = IQR; whiskers = 1.5 × IQR. *** *p* < 0.001 (Mann–Whitney U with Bonferroni correction, adjusted alpha = 0.0167). MPV = mean platelet volume; PDW = platelet distribution width; P-LCR = platelet large cell ratio. ns: not significant.

Consumption-like patients had higher MPV (12.1 vs. 10.5 fL), PDW (16.0 vs. 11.9 fL), and P-LCR (41.2% vs. 30.6%) than production-like patients—all differences *p* < 0.001 after Bonferroni correction, with large effect sizes (PDW r = 0.798, P-LCR r = 0.961). PCT differed only marginally (r = 0.149), suggesting that platelet size rather than total count distinguishes the two phenotypes (Table 2; Figure 2).

In the 263 of 703 thrombocytopenic patients for whom P-LCR data were available, P-LCR alone (not used in phenotype definition) identified the consumption-like phenotype with AUC = 0.981 at a cut-off of 35.3% (sensitivity 95.4%, specificity 91.0%). Because P-LCR was not part of the phenotype definition, this result is not circular: the consumption-like phenotype was defined solely by MPV, yet an independent index (P-LCR) identifies the same grouping with high accuracy, consistent with the MPV-based classification reflecting a genuine biological distinction rather than a tautological one—though it does not constitute formal validation. Given that 62.6% of P-LCR values are missing among thrombocytopenic patients, the finding cannot be generalized to the full cohort.

### 3.3. Mortality Across Phenotypic Groups

Mortality differed across phenotypic groups (chi-square *p* = 0.010). Consumption-like patients had the highest observed rate (52.0%), followed by production-like (46.7%) and PLT-normal (42.4%). On pairwise testing, only the consumption-like vs. PLT-normal comparison reached significance (Fisher’s exact *p* = 0.006); production-like versus PLT-normal did not (*p* = 0.116). The ordering should not be read as a dose–response gradient. Only the consumption-like versus PLT-normal difference was statistically supported. Subgroup data are in Table 3.

### 3.4. Multivariable Logistic Regression

In the multivariable model (*n* = 1954; AUC = 0.678), consumption-like thrombocytopenia was independently associated with ICU death (OR 1.46, 95% CI 1.08–1.99; *p* = 0.016). Production-like thrombocytopenia was not (OR 1.12, 95% CI 0.88–1.43; *p* = 0.302). The remaining significant predictors were APACHE II (OR 1.04 per point), malignancy (OR 2.65), and hypertension (OR 1.26; *p* = 0.032). The model showed good calibration (Hosmer–Lemeshow χ^2^ = 8.81, df = 8; *p* = 0.359). Full results are in Table 4.

When PLT, MPV, and PDW were entered as separate continuous variables instead of the phenotype label, none reached significance (*p* = 0.201, 0.531, and 0.498, respectively). The combined label may capture a signal that the individual components lose when analyzed separately; collinearity between PLT and MPV is an alternative explanation.

### 3.5. Sensitivity Analysis: Hemogram-Derived Inflammatory Indices

None of the five hemogram-derived indices—NLR, LMR, PLR, RDW-CV, or SII—was associated with ICU mortality in this cohort (all *p* > 0.36; rank-biserial r < 0.03 for each). PLR, RDW, and SII differed across phenotype groups (Kruskal–Wallis, *p* < 0.001), as expected: PLR declines mechanically when platelet counts are low, and RDW is higher in the production-like group, consistent with concurrent erythropoietic stress rather than an independent mortality signal. When NLR and RDW were added to the primary model, the adjusted OR for consumption-like thrombocytopenia was 1.49 (95% CI 1.10–2.03; *p* = 0.011)—essentially unchanged from the base estimate of 1.46. Neither NLR (OR 1.00; *p* = 0.316) nor RDW (OR 0.99; *p* = 0.427) reached significance. Model AUC did not change (0.680 vs. 0.681). A likelihood ratio test confirmed that NLR and RDW together added no explanatory value (χ^2^ = 1.57, df = 2; *p* = 0.457). Spearman correlations between NLR, RDW, and the primary model covariates were all ≤0.12, consistent with negligible confounding. Detailed results are in Supplementary Table S3 and Figure S1.

### 3.6. Malignancy Subgroup Analysis

**Figure 3:**
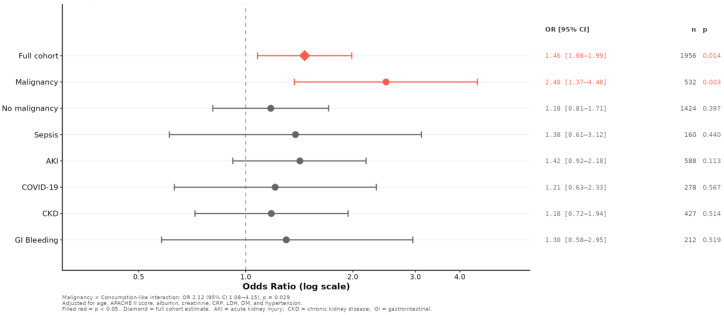
Forest plot of adjusted odds ratios for consumption-like thrombocytopenia and ICU mortality across prespecified subgroups. Adjusted for age, APACHE II score, albumin, creatinine, CRP, LDH, diabetes mellitus, and hypertension. Filled symbols (red) indicate *p* < 0.05. Diamond = full cohort estimate. The malignancy × consumption-like phenotype interaction OR was 2.12 (95% CI 1.08–4.15; *p* = 0.029). AKI = acute kidney injury; CKD = chronic kidney disease; CRP = C-reactive protein; DM = diabetes mellitus; GI = gastrointestinal; LDH = lactate dehydrogenase; OR = odds ratio; CI = confidence interval.

**Figure 4:**
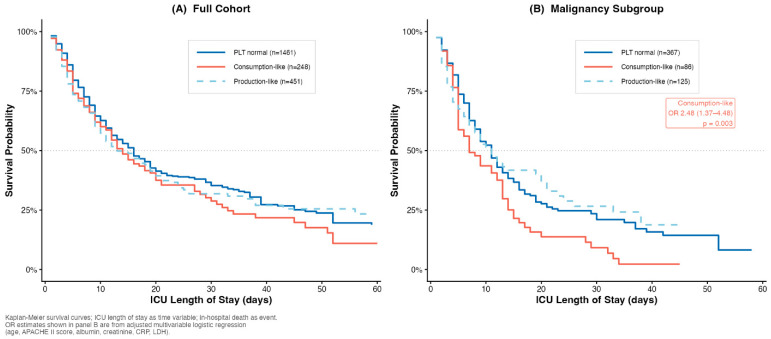
Kaplan–Meier survival curves by platelet phenotype for (**A**) the full cohort and (**B**) the malignancy subgroup. Time variable = ICU length of stay; event = in-hospital death. OR estimates shown in panel B are from adjusted multivariable logistic regression (age, APACHE II score, albumin, creatinine, CRP, and LDH). ICU = intensive care unit; OR = odds ratio; CRP = C-reactive protein; LDH = lactate dehydrogenase.

Among the 585 patients with malignancy, mortality was 76.7% in the consumption-like group, compared with 58.6% in PLT-normal (*p* = 0.002) and 56.8% in production-like patients (*p* = 0.003). In the malignancy-restricted multivariable model (*n* = 531), the adjusted OR for the consumption-like phenotype was 2.48 (95% CI 1.37–4.48; *p* = 0.003), higher than the full-cohort estimate of 1.46 (Figure 3 and Figure 4). Production-like thrombocytopenia was not associated with mortality in this subgroup (OR 1.25). In contrast, among the 1419 patients without malignancy, consumption-like thrombocytopenia was not independently associated with ICU death (OR 1.18, 95% CI 0.82–1.74; *p* = 0.397). A formal interaction test supported effect modification: the malignancy × consumption-like phenotype interaction term had an OR of 2.12 (95% CI 1.08–4.15; *p* = 0.029). The confidence interval just excluded 1.0, and this finding should be interpreted cautiously until replicated.

### 3.7. Other Admission Diagnoses

Across the remaining subgroups, AKI (*n* = 588), gastrointestinal bleeding (*n* = 212), sepsis (*n* = 160), COVID-19 (*n* = 278), and CKD (*n* = 427); consumption-like patients had numerically higher mortality, but none of the individual comparisons reached significance. Sample sizes were small, so these results are uninformative rather than reassuring. The directional consistency is noted but should not be interpreted as confirmatory (Table 3).

## 4. Discussion

The full-cohort signal was modest: OR 1.46, significant but with a wide confidence interval. In patients with malignancy, the estimate was higher, OR 2.48, and the interaction term reached statistical significance (OR 2.12; *p* = 0.029), though barely. Among patients without malignancy, no association was found (OR 1.18; *p* = 0.397). One caveat on the sequence is that malignancy was a prespecified subgroup, but the interaction test was added after we observed the divergence. This limits what can be inferred, and the finding needs prospective confirmation before it can carry any clinical weight.

Thrombocytopenia predicts ICU admission and death in patients with malignancy [11], a population in which mortality risk extends into subsequent admissions [12]. Ferreyro et al. [11], in a population-based cohort of 87,965 patients with hematologic malignancy, found that baseline thrombocytopenia independently predicted ICU admission—but, as in almost every other study, they examined only platelet count. What this dataset adds is a pathophysiological dimension, framed as a surrogate phenotype rather than a confirmed mechanism. Among thrombocytopenic ICU patients, those with elevated MPV had higher mortality than those with low or normal MPV. The production-like group had lower median platelet counts (71 vs. 93 × 10^9^/L) yet lower mortality. This reversal is not explicable by platelet count alone, and it is the pattern that the phenotypic classification is built to detect.

P-LCR was not used to define the phenotypes. Among the 263 thrombocytopenic patients with available P-LCR data, it identified the consumption-like phenotype with an AUC of 0.981 at a cut-off of 35.3%, a high value, though the subset covers only 37.4% of thrombocytopenic patients, and its representativeness is unknown. The result is consistent with the two groups having different underlying platelet biology. It is not independent external validation.

Why should the consumption-like phenotype matter specifically in malignancy? One possible explanation is that malignant cells express tissue factor and shed procoagulant microparticles, driving low-grade DIC that consumes platelets and stimulates thrombopoiesis, raising MPV. In septic ICU patients, elevated MPV has similarly been associated with higher mortality and greater disease severity [13,14,15,16], consistent with peripheral platelet consumption as a shared adverse signal across different clinical contexts—though the drivers likely differ from the malignancy-related pathway described here. This mechanism is less prominent when thrombocytopenia reflects impaired marrow production, which may explain the weaker prognostic weight of the production-like phenotype despite its lower platelet counts. Outside malignancy, ICU thrombocytopenia has many causes—sepsis, drugs, dilution, hypersplenism—and MPV elevation is less specific for consumption in that context. Whether that heterogeneity accounts for the absence of a non-malignancy signal is speculative.

When PLT, MPV, and PDW were modeled as separate continuous variables, none reached significance. The combined phenotype label may capture a signal that the individual indices lose when analyzed in isolation, or collinearity between PLT and MPV may simply attenuate both. We cannot distinguish these explanations with the current data.

One question the data allow us to address is whether the consumption-like phenotype signal is a proxy for systemic inflammation, as captured by routine leukocyte-derived indices. NLR has been associated with mortality in unselected ICU populations [17], though not consistently in sepsis subgroups [17]. In our cohort, none of NLR, LMR, PLR, RDW, or SII was associated with ICU death, and adding NLR and RDW to the primary model left the consumption-like OR essentially unchanged (1.49 vs. 1.46) with no improvement in model fit (LRT *p* = 0.457). The phenotype signal appears to reflect something the standard inflammatory ratios do not capture—most likely the platelet-specific biology of peripheral consumption rather than a nonspecific inflammatory state.

Several limitations should be noted. This is a single-center retrospective study. The MPV threshold was derived from our institutional reference range and may not apply to centers using different analyzers. P-LCR was missing in 62.6% of thrombocytopenic patients. We had no bone marrow data, reticulated platelet counts, D-dimer, or fibrinogen—variables that would have allowed direct verification of the phenotypic assignments. Important ICU-related confounders, including vasopressor use, mechanical ventilation, renal replacement therapy, transfusion history, coagulation status, and chemotherapy exposure, were not available and therefore not included in the regression model. Residual confounding from these variables cannot be excluded, and the reported associations should not be interpreted as causal. The model AUC was 0.678, modest but not surprising given how difficult ICU mortality is to predict. APACHE II underestimated mortality by roughly 18% across the full cohort (SMR 1.18), with the excess concentrated in patients with malignancy (SMR 1.49), consistent with known APACHE II limitations in oncological populations [18,19]. Whether acting on the platelet phenotype at admission would change outcomes is unknown.

Two findings stand out. In the full cohort, consumption-like thrombocytopenia was associated with a 46% increase in adjusted odds of ICU death—statistically significant and detectable from the standard blood count alone. In patients with malignancy, the OR rose to 2.48, and the interaction term reached statistical significance, though narrowly (*p* = 0.029). Whether this reflects a genuine mechanistic pathway or is chance amplification of a modest signal requires a prospective oncological ICU cohort to determine. A cancer patient admitted with thrombocytopenia and an elevated MPV may carry a higher risk than severity scores alone suggest. Whether recognizing that risk changes management is a question this dataset cannot answer.

## Figures and Tables

**Table 1:** Baseline Characteristics by Platelet Phenotype Group.

Variable	PLT Normal (*n* = 1471)	Consumption-Like (*n* = 249)	Production-Like (*n* = 454)	*p* Value
**Age, years, median (IQR)**	70 (60–78)	70 (62–77)	67 (57–76)	0.002
Male sex, *n* (%)	848 (57.6%)	141 (56.6%)	266 (58.6%)	0.876
**Continuous variables—median (IQR)**				
APACHE II score	18 (10–26)	18.5 (10–28)	18 (10–28)	0.579
GCS score	14 (10–15)	14 (11–15)	13 (9.5–15)	0.200
PLT (×10^9^/L)	262 (207–342)	93 (56–121)	71 (32–109)	<0.001
MPV (fL)	10.5 (9.8–11.3)	12.1 (11.8–12.7)	10.5 (10.1–10.9)	<0.001
PDW (fL)	11.9 (10.5–13.7)	16.0 (14.1–18.5)	11.9 (10.9–13.2)	<0.001
P-LCR (%)	28.6 (23–35)	41.2 (38–46)	30.6 (26–33)	<0.001
PCT	0.30 (0.20–0.30)	0.11 (0.07–0.15)	0.10 (0.07–0.13)	<0.001
WBC (×10^9^/L)	13.0 (9.5–18.4)	9.6 (4.9–14.4)	8.1 (3.7–14.7)	<0.001
Hemoglobin (g/dL)	10.4 (8.8–12.1)	9.4 (8.0–10.7)	8.9 (7.6–10.2)	<0.001
RDW-CV (%)	15.7 (14.1–17.9)	16.3 (14.7–17.6)	17.2 (15.2–20.2)	<0.001
CRP (mg/L)	161 (79–272)	151 (89–294)	149 (84–242)	0.325
LDH (U/L)	280 (207–421)	313 (230–513)	371 (228–811)	<0.001
Albumin (g/L)	30.2 (25.5–35.6)	26.0 (21.7–30.4)	26.0 (21.7–31.6)	<0.001
Creatinine (mg/dL)	1.3 (0.8–2.9)	2.0 (1.0–3.7)	1.6 (0.9–2.8)	<0.001
ICU LOS (days)	7 (4–13)	8 (4–14)	6 (3–12)	0.113
**Comorbidities, *n* (%)**				
Diabetes mellitus	544 (37.0%)	91 (36.5%)	153 (33.7%)	0.443
Hypertension	915 (62.2%)	141 (56.6%)	275 (60.6%)	0.236
Chronic kidney disease	286 (19.4%)	43 (17.3%)	98 (21.6%)	0.366
Coronary artery disease	344 (23.4%)	49 (19.7%)	107 (23.6%)	0.415
COPD	126 (8.6%)	20 (8.0%)	41 (9.0%)	0.900
Stroke	217 (14.8%)	32 (12.9%)	50 (11.0%)	0.118
Heart failure	248 (16.9%)	42 (16.9%)	76 (16.7%)	0.998
Malignancy	370 (25.2%)	86 (34.5%) *	125 (27.5%)	0.008
COVID-19	196 (13.3%)	24 (9.6%)	58 (12.8%)	0.273
Intubation	113 (7.7%)	19 (7.6%)	30 (6.6%)	0.743
ICU Mortality, *n* (%)	620 (42.4%)	129 (52.0%) †	211 (46.7%)	0.010

IQR: interquartile range; PLT: platelet count; MPV: mean platelet volume; PDW: platelet distribution width; P-LCR: platelet-large cell ratio; PCT: plateletcrit; WBC: white blood cell count; RDW-CV: red cell distribution width; CRP: C-reactive protein; LDH: lactate dehydrogenase; ICU LOS: intensive care unit length of stay. * *p* = 0.008 vs. PLT normal (chi-square); † *p* = 0.006 vs. PLT normal (Fisher’s exact test).

**Table 2:** Platelet Index Profile in Thrombocytopenic Patients (*n* = 703): Consumption-like vs. Production-like.

Index	Consumption-Like Median (IQR)	Production-Like Median (IQR)	AUC *	Effect Size r	*p* Value
**MPV (fL)**	12.1 (11.8–12.7)	10.5 (10.1–10.9)	1.000 †	1.000	<0.001
**PDW (fL)**	16.0 (14.1–18.5)	11.9 (10.9–13.2)	0.899	0.798	<0.001
**P-LCR (%)**	41.2 (38–46)	30.6 (26–33)	0.981 ‡	0.961	<0.001
**PCT**	0.11 (0.07–0.15)	0.10 (0.07–0.13)	0.575	0.149	0.005

MPV: mean platelet volume; PDW: platelet distribution width; P-LCR: platelet-large cell ratio; PCT: plateletcrit; r: rank-biserial correlation (|r| > 0.5 = large effect). * AUC for discriminating consumption-like from production-like within thrombocytopenic patients. † MPV AUC = 1.000 by construction (phenotype defined partly on MPV). ‡ P-LCR independent validation (not used in phenotype definition); available in 263 of 703 thrombocytopenic patients.

**Table 3:** ICU Mortality by Platelet Phenotype in Clinical Subgroups.

Subgroup	*n*	PLT Normal	Consumption-Like	Production-Like	*p* Value
Overall	2188	42.4%	52.0% *	46.7%	0.010
Malignancy	585	58.6%	76.7% **	56.8%	0.005
Acute kidney injury	588	47.5%	58.6%	50.4%	0.230
Pneumonia	473	47.2%	59.5%	51.3%	0.180
Gastrointestinal bleeding	212	32.0%	45.0%	26.7%	0.343
Sepsis	160	55.6%	63.6%	51.3%	0.647
COVID-19	278	39.3%	33.3%	46.6%	0.470
Chronic kidney disease	427	55.6%	55.8%	60.2%	0.724

* *p* = 0.006 vs. PLT normal; ** *p* = 0.002 vs. PLT normal, *p* = 0.003 vs. production-like (Fisher’s exact test).

**Table 4:** Multivariable Logistic Regression for ICU Mortality (*n* = 1954; AUC = 0.678).

Variable	OR	95% CI	*p* Value
APACHE II score (per point)	1.037	1.029–1.045	<0.001
Malignancy (yes vs. no)	2.649	2.135–3.287	<0.001
Consumption-like thrombocytopenia	1.464	1.079–1.985	0.016
Production-like thrombocytopenia	1.124	0.881–1.433	0.302
Age (per year)	0.999	0.993–1.005	0.794
Albumin (per g/L)	1.008	0.994–1.022	0.275
Creatinine (per mg/dL)	0.980	0.934–1.028	0.412
CRP (per mg/L)	1.000	0.999–1.001	0.840
LDH (per U/L)	1.000	1.000–1.000	0.471
Diabetes mellitus	1.112	0.903–1.369	0.318
Hypertension	1.255	1.020–1.544	0.032

Reference: PLT normal (platelet count ≥ 150 × 10^9^/L). OR: odds ratio; CI: confidence interval; AUC: area under the ROC curve.

## Data Availability

The data presented in this study are available on request from the corresponding author. The data are not publicly available due to institutional privacy policies and participant confidentiality agreements.

## Supplementary Materials

The following supporting information can be downloaded at: https://www.mdpi.com/article/10.3390/jcm15124720/s1, Table S1: Sensitivity Analysis: Effect of Alternative MPV Thresholds on Consumption-like Thrombocytopenia—ICU Mortality Association; Table S2: Missing Data Summary for Primary Analysis Variables (N = 2188); Table S3: Hemogram-derived Inflammatory Indices by Platelet Phenotype Group; Table S4: APACHE II-Predicted versus Observed ICU Mortality and Standardized Mortality Ratio (SMR) by Score Category and Malignancy Status; Figure S1: Adjusted Odds Ratios: Base Model versus Sensitivity Analysis (+NLR, +RDW).

## References

[1] Pene F., Russell L., Aubron C. (2025). Thrombocytopenia in the intensive care unit: Diagnosis and management. Ann. Intensive Care.

[2] Jonsson A.B., Rygård S.L., Hildebrandt T., Perner A., Møller M.H., Russell L. (2021). Thrombocytopenia in intensive care unit patients: A scoping review. Acta Anaesthesiol. Scand..

[3] van Wonderen S.F., Raasveld S.J., Flint A.W.J., Schenk J., van den Oord C., Reuland M.C., de Bruin S., Bakker J., Cecconi M., Feldheiser A. (2025). Platelet Transfusion Practices in the ICU: A Prospective Multicenter Cohort Study. Crit. Care Med..

[4] Zarychanski R., Houston D.S. (2017). Assessing thrombocytopenia in the intensive care unit: The past, present, and future. Hematol. Am. Soc. Hematol. Educ. Program.

[5] Knöbl P. (2016). Thrombocytopenia in the intensive care unit: Diagnosis, differential diagnosis, and treatment. Med. Klin. Intensivmed. Notfmed..

[6] Jiang X., Zhang W., Ma X., Cheng X. (2022). Risk of hospital mortality in critically ill patients with transient and persistent thrombocytopenia: A retrospective study. Shock.

[7] Ali U., Chopra M., Knight G. (2025). Trajectories of platelet indices and their association with mortality in the ICU-a longitudinal cohort study. Scand. J. Clin. Lab. Investig..

[8] Chen J., Gao X., Shen S., Xu J., Sun Z., Lin R., Dai Z., Su L., Christiani D.C., Chen F. (2022). Association of longitudinal platelet count trajectory with ICU mortality: A multi-cohort study. Front. Immunol..

[9] Li J., Li R., Jin X., Ren J., Zhang J., Gao Y., Hou Y., Zhang X., Wang G. (2025). Platelet count trajectory patterns and prognosis in critically ill patients with thrombocytopenia: Based on latent growth mixture model analysis. Thromb. Res..

[10] Grossi A., Vannucchi A.M., Casprini P., Guidi S., Rafanelli D., Pecchioli M.G., Ferrini P.R. (1983). Different patterns of platelet turnover in chronic idiopathic thrombocytopenic purpura. Scand. J. Haematol..

[11] Ferreyro B.L., Scales D.C., Wunsch H., Cheung M.C., Gupta V., Saskin R., Thyagu S., Munshi L. (2021). Critical illness in patients with hematologic malignancy: A population-based cohort study. Intensive Care Med..

[12] AbuSara A.K., Nazer L.H., Hawari F.I. (2019). ICU readmission of patients with cancer: Incidence, risk factors and mortality. J. Crit. Care.

[13] Vardon-Bounes F., Ruiz S., Gratacap M.P., Garcia C., Payrastre B., Minville V. (2019). Platelets are critical key players in sepsis. Int. J. Mol. Sci..

[14] Fogagnolo A., Taccone F.S., Benetto G., Franchi F., Scolletta S., Cotoia A., Kozhevnikova I., Volta C.A., Spadaro S. (2021). Platelet morphological indices on Intensive Care Unit admission predict mortality in septic but not in non-septic patients. Minerva Anestesiol..

[15] Vélez-Páez J.L., Legua P., Vélez-Páez P., Irigoyen E., Andrade H., Jara A., López F., Pérez-Galarza J., Baldeón L. (2022). Mean platelet volume and mean platelet volume to platelet count ratio as predictors of severity and mortality in sepsis. PLoS ONE.

[16] Vardon-Bounes F., Gratacap M.P., Groyer S., Ruiz S., Georges B., Seguin T., Garcia C., Payrastre B., Conil J.-M., Minville V. (2019). Kinetics of mean platelet volume predicts mortality in patients with septic shock. PLoS ONE.

[17] Salciccioli J.D., Marshall D.C., Pimentel M.A., Santos M.D., Pollard T., Celi L.A., Shalhoub J. (2015). The association between the neutrophil-to-lymphocyte ratio and mortality in critical illness: An observational cohort study. Crit. Care.

[18] de Vries V.A., Müller M.C.A., Sesmu Arbous M., Biemond B.J., Blijlevens N.M.A., Kusadasi N., Choi G.C.W., Vlaar A.P.J., van Westerloo D.J., Kluin-Nelemans H.C. (2018). Time trend analysis of long term outcome of patients with haematological malignancies admitted at dutch intensive care units. Br. J. Haematol..

[19] Cabrera Losada A., Correa Oviedo M.A., Herrera Villazón V.C., Gil-Tamayo S., Molina C.F., Vich C.G.-E., Estrada V.H.N. (2024). Towards better mortality prediction in cancer patients in the ICU: A comparative analysis of prognostic scales: Systematic literature review. Med. Intensiva (Engl. Ed.).

