# Supplementary material for: Consumption-like Thrombocytopenia Phenotype Predicts ICU Mortality with Particular Relevance in Patients with Malignancy: A Retrospective Single-Center Cohort Study of 2188 Patients
Source: J Clin Med. 2026 Jun 17;15(12):4720. doi: 10.3390/jcm15124720 (PMC13302326; doi:10.3390/jcm15124720)
Supplement: Supplementary file 1 [file jcm-15-04720-s001.zip › jcm-4327367-supplementary.pdf]

SUPPLEMENTARY MATERIALS

Supplementary Table S1. Sensitivity Analysis: Effect of Alternative MPV Thresholds on Consumption-like Thrombocytopenia – ICU Mortality Association

MPV Threshold	Consumption-like n (% of thrombocytopenic)	Adjusted OR	95% CI	p value
50th percentile (10.5 fL)	359 (51.1%)	1.09	0.84–1.41	0.522
**75th percentile (11.3 fL) — primary analysis**	**249 (35.4%)**	**1.46**	**1.08–1.99**	**0.014**
Upper reference limit (12.0 fL)	134 (19.1%)	1.44	0.98–2.13	0.064

Adjusted for age, APACHE II, albumin, creatinine, CRP, LDH, malignancy, hypertension, and platelet phenotype (production-like vs. PLT-normal). ORs are for consumption-like thrombocytopenia vs. PLT-normal. The 75th-percentile threshold (primary analysis) showed the strongest and most consistent association. The upper reference limit (12.0 fL) corresponds to the 90th percentile of the non-thrombocytopenic population in this cohort. OR, odds ratio; CI, confidence interval; fL, femtolitres; PLT, platelet count; MPV, mean platelet volume.

Supplementary Table S2. Missing Data Summary for Primary Analysis Variables (N=2,188)

Variable	N missing	% missing
Age	0	0.0%
APACHE II score	7	0.3%
Albumin	40	1.8%
Creatinine	12	0.5%
CRP	0	0.0%
LDH	204	9.3%
MPV (used for phenotype classification)	273	12.5%
P-LCR (full cohort)	1,308	59.8%
P-LCR (thrombocytopenic patients only, n=703)	440	62.6%
Primary outcome (death)	12	0.5%

Most variables had <2% missingness. LDH (9.3%) and MPV (12.5%) were the primary sources of incomplete data. All analyses used complete cases. Patients with missing MPV were excluded from phenotype classification and, therefore, from the primary analysis. The complete-case model (n=1,954) was compared against the full eligible cohort (n=2,188); the 234 excluded patients did not differ significantly from included patients in age, sex, or APACHE II score distribution, consistent with missing-at-random rather than informative missingness.

**Supplementary Table S3. Hemogram-derived Inflammatory Indices by Platelet Phenotype Group**					
	**Overall (n=2,188)**	**PLT-normal (n=1,471)**	**Consumption-like (n=249)**	**Production-like (n=454)**	**p value**
**NLR**	9.78 [4.93, 19.12]	10.36 [5.59, 19.50]	9.46 [4.13, 18.77]	7.70 [2.90, 16.46]	<0.001
**LMR**	1.56 [0.93, 2.73]	1.53 [0.93, 2.55]	1.76 [0.88, 2.95]	1.60 [0.93, 3.45]	0.057
**PLR***	206.49 [110.80, 368.33]	253.68 [155.60, 428.00]	121.85 [68.33, 213.03]	100.00 [44.61, 214.56]	<0.001
**RDW-CV***	16.00 [14.40, 18.30]	15.70 [14.10, 17.90]	16.30 [14.70, 17.60]	17.20 [15.20, 20.20]	<0.001
**SII***	1867.91 [783.73, 4327.14]	2813.35 [1408.88, 5450.36]	866.34 [340.24, 1808.96]	519.14 [119.93, 1389.10]	<0.001
**WBC**	11.87 [8.02, 17.25]	13.02 [9.50, 18.43]	9.64 [4.92, 14.41]	8.06 [3.65, 14.73]	<0.001
**Hgb**	9.90 [8.40, 11.70]	10.40 [8.80, 12.10]	9.40 [8.00, 10.70]	8.90 [7.60, 10.20]	<0.001

*All values are median [IQR]; p values from Kruskal-Wallis test. *Differences in PLR, RDW-CV, and SII across phenotype groups reflect their mathematical dependence on platelet count and are not interpreted as independent mortality associations. NLR: neutrophil-to-lymphocyte ratio; LMR: lymphocyte-to-monocyte ratio; PLR: platelet-to-lymphocyte ratio; RDW-CV: red cell distribution width; SII: systemic immune-inflammation index; WBC: white blood cell count; Hgb: hemoglobin.*

Supplementary Table S4. APACHE II–Predicted versus Observed ICU Mortality and Standardized Mortality Ratio (SMR) by Score Category and Malignancy Status

APACHE II–predicted mortality was calculated for each patient using the standard medical non-operative formula: logit(p) = −3.517 + 0.146 × APACHE II score; predicted probability = 1 / (1 + e⁻logit). Standardized mortality ratio (SMR) = observed mortality / mean predicted mortality. 95% CI for SMR was derived using the Poisson approximation: SMR × (1 ± 1.96 / √(9 × observed deaths)). APACHE II score available in 2,181 of 2,188 patients (99.7%).

Group	n	Predicted Mortality	Observed Mortality	SMR	SMR 95% CI
**Overall cohort**	2,181	37.5%	44.4%	**1.18**	1.155–1.205
APACHE II <10	527	6.4%	40.1%	6.24	—
APACHE II 10–14	258	14.5%	32.6%	2.24	—
APACHE II 15–19	432	26.1%	33.9%	1.30	—
APACHE II 20–24	324	42.4%	36.2%	0.86	—
APACHE II 25–29	190	60.2%	55.1%	0.92	—
APACHE II ≥30	450	85.1%	67.9%	0.80	—
**Malignancy subgroup**	584	41.3%	61.3%	**1.49**	—
**Non-malignancy subgroup**	1,597	36.1%	38.2%	**1.06**	—

SMR = standardized mortality ratio; CI = confidence interval. SMR >1.0 indicates observed mortality exceeds APACHE II prediction; SMR <1.0 indicates prediction exceeds observed. Individual-category 95% CIs are not shown due to low event counts in some cells. APACHE II is known to underestimate mortality in patients with malignancy; for methodological basis, see de Vries et al. (Br J Haematol 2018) and Cabrera Losada et al. (Med Intensiva 2024).

Figure S1. Adjusted Odds Ratios: Base Model versus Sensitivity Analysis (+NLR, +RDW)

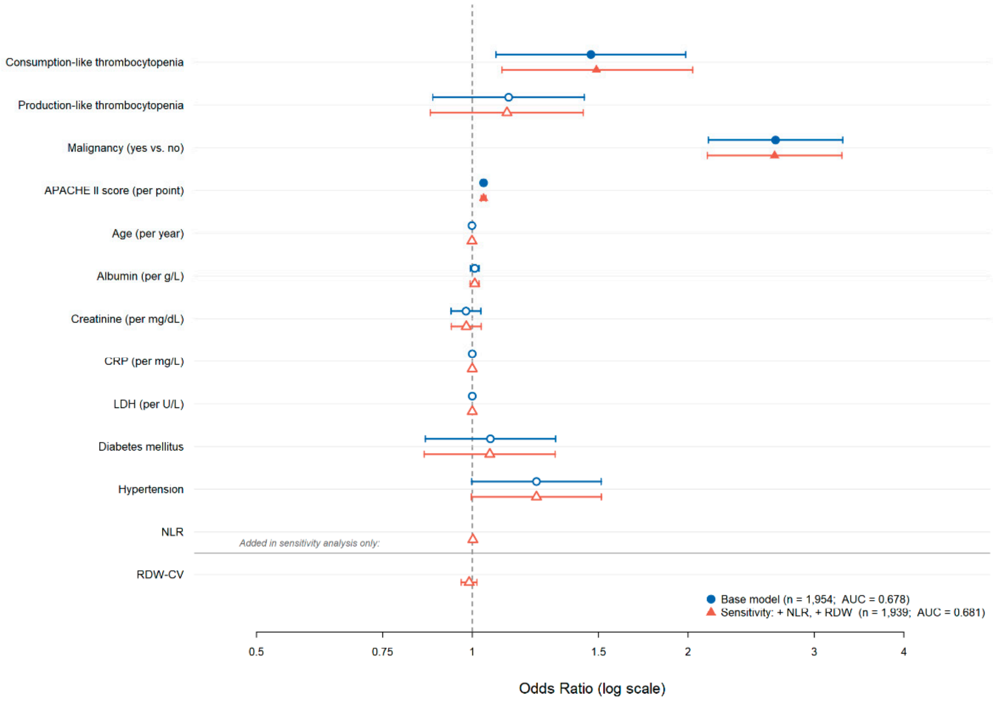

Forest plot of adjusted odds ratios from the base multivariable model (blue circles) and the sensitivity model with NLR and RDW added (red triangles). Point estimates with 95% confidence intervals on a log scale. Neither NLR nor RDW reached significance in the sensitivity model (both p > 0.30), and the consumption-like thrombocytopenia estimate remained unchanged. Base model: n=1,954, AUC=0.678; sensitivity model: n=1,939, AUC=0.681. NLR: neutrophil-to-lymphocyte ratio; RDW: red cell distribution width.
